# Tep1 Regulates Yki Activity in Neural Stem Cells in *Drosophila* Glioma Model

**DOI:** 10.3389/fcell.2020.00306

**Published:** 2020-05-08

**Authors:** Karishma Gangwani, Kirti Snigdha, Madhuri Kango-Singh

**Affiliations:** ^1^Department of Biology, University of Dayton, Dayton, OH, United States; ^2^Center for Tissue Regeneration and Engineering at Dayton (TREND), University of Dayton, Dayton, OH, United States; ^3^Premedical Programs, University of Dayton, Dayton, OH, United States; ^4^Integrated Science and Engineering Center (ISE), University of Dayton, Dayton, OH, United States

**Keywords:** Yki, Tep, neural stem cell, glioma, *Drosophila*, Hippo pathway, Tep1

## Abstract

Glioblastoma Multiforme (GBM) is the most common form of malignant brain tumor with poor prognosis. Amplification of Epidermal Growth Factor Receptor (EGFR), and mutations leading to activation of Phosphatidyl-Inositol-3 Kinase (PI3K) pathway are commonly associated with GBM. Using a previously published *Drosophila* glioma model generated by coactivation of PI3K and EGFR pathways [by downregulation of Pten and overexpression of oncogenic Ras] in glial cells, we showed that the *Drosophila* Tep1 gene (ortholog of human CD109) regulates Yki (the *Drosophila* ortholog of human YAP/TAZ) via an evolutionarily conserved mechanism. Oncogenic signaling by the YAP/TAZ pathway occurs in cells that acquire CD109 expression in response to the inflammatory environment induced by radiation in clinically relevant models. Further, downregulation of Tep1 caused a reduction in Yki activity and reduced glioma growth. A key function of Yki in larval CNS is stem cell renewal and formation of neuroblasts. Other reports suggest different upstream regulators of Yki activity in the optic lobe versus the central brain regions of the larval CNS. We hypothesized that Tep1 interacts with the Hippo pathway effector Yki to regulate neuroblast numbers. We tested if Tep1 acts through Yki to affect glioma growth, and if in normal cells Tep1 affects neuroblast number and proliferation. Our data suggests that Tep1 affects Yki mediated stem cell renewal in glioma, as reduction of Tep significantly decreases the number of neuroblasts in glioma. Thus, we identify Tep1-Yki interaction in the larval CNS that plays a key role in glioma growth and progression.

## Introduction

Glioblastoma multiforme (GBM) is a primary malignant adult brain tumor with extremely poor prognosis (Ostrom et al., 2017). Current therapies such as surgery and chemo- and/or radiotherapy only provide palliative care resulting in median GBM patient survival of about 14–15 months (Ghinda et al., 2016). Using patient samples, efforts to identify underlying genetic aberrations that lead to GBM have resulted in the identification of genomic amplification of EGFR, PDGFRA, and FGFR; along with activating mutations and overexpression of Receptor Tyrosine Kinases (RTKs) and genes of the PI3K pathway. Additional mutations include inactivation of p53/RB pathways (Singh et al., 2004). Of these, amplification of EGFR is the most frequent alteration, followed by activating mutations in PI3K [PI3K^CA^] and loss of PTEN [phosphatidyl-inositol 3-phosphate (PIP3) lipid phosphatase] (Gao et al., 2000). These alterations result in constitutive activation of PI3K (Read et al., 2009). Taking advantage of the conservation of genes of the EGFR and PI3K pathways, preclinical glioma models have been made in *Drosophila melanogaster* – a powerful *in-vivo* genetic model system (Gao et al., 2000; Read, 2011; Waghmare et al., 2014; Cheng et al., 2016; Chen and Read, 2019). Also, the genes and proteins required for neural development perform identical functions resulting in the presence of analogous cell types in fly and human central nervous system (CNS) (Karim et al., 1996; Voas and Rebay, 2004; Freeman and Doherty, 2006; Furnari et al., 2007; Wilson et al., 2010; Homem et al., 2015).

**FIGURE 1:**
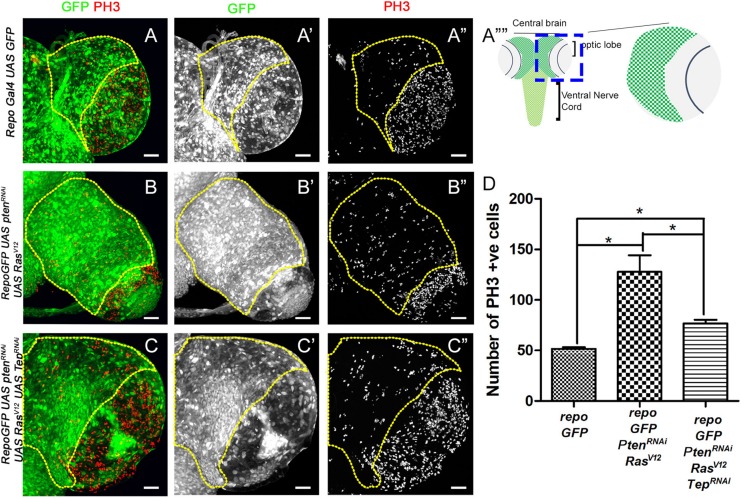
Loss of Tep1 reduces the mitotic index in glioma. Comparisons of PH3 positive cell numbers in the central brain region of *Drosophila* third instar larval brain (see, schematic in **A””**) are shown for the following genotypes: wild type control *repo*-Gal4>GFP (**A–A”**, referred to as *repo-GFP* in all subsequent panels and figures), and glioma from *repo*-GFP>*Pten*^RNAi^; *UAS-Ras*^V12^ **(B–B”)**, and *repo*-GFP>*Pten*^RNAi^; *UAS-Ras*^V12^; *UAS-Tep1*^RNAi^ **(C–C”)**. Glial cells are marked by GFP (green, gray) and PH3 (red, gray). **(D)** Quantification of number of PH3 positive cells in the central brain is shown in the graph. Unpaired 2-tailed *T*-test with *n* = 5, 95% confidence was performed using GraphPad Prism 5, *p* < 0.05. Yellow ROI boxes indicate area in which PH3 positive cells are counted. All images were scanned at identical magnification [20×, scale bars, 100 μm = 62px].

In *Drosophila*, the larval CNS is comprised of two brain lobes and a ventral nerve cord. All neurons project their axons and dendrites into the central neuropil which harbors all synapses (Sprecher et al., 2011; Courgeon and Desplan, 2019). There are two types of progenitor or neural stem cells (NSCs aka neuroblasts) in the *Drosophila* larval CNS – the optic lobe (OL) neuroepithelium and the central brain (CB) neuroblasts (Figure 1A””). The neuroepithelium within the OL gives rise to medulla neuroblasts, whereas in the CB and ventral nerve cord (VNC) the neuroblasts of Type I and Type II lineages are found (Bello et al., 2008; Boone and Doe, 2008). During brain development, NSCs undergo self-renewing asymmetric cell divisions to produce a neuroblast and a smaller daughter cell, the ganglion mother cell (GMC) that divides once more to differentiate into neuron or glia (Freeman and Doherty, 2006; Homem and Knoblich, 2012; Homem et al., 2015). To ensure that the correct number and type of neurons are made, NSCs must coordinate cell cycle entry and exit with a strict developmental timing. Deregulation of these NSCs could give rise to developmental defects like microcephaly, or overgrowth of the brain associated with the formation of tumors (Betschinger et al., 2006; Choksi et al., 2006; Wang et al., 2006; Bowman et al., 2008).

The Hippo pathway is a major regulator of organ size and is comprised of several upstream regulators that modify the activity of a core kinase cassette to control the downstream transcriptional effector Yorkie (Yki, *Drosophila* homolog of mammalian YAP/TAZ) (Kango-Singh and Singh, 2009; Grusche et al., 2010; Halder and Johnson, 2011; Snigdha et al., 2019; Zheng and Pan, 2019). The Hippo pathway is known to regulate the proliferation of two cell populations in the larval *Drosophila* brain: glia and the neuroepithelium (Reddy et al., 2010; Reddy and Irvine, 2011). The pathway restricts neuroblast proliferation potential and neuronal cell number to regulate brain size during the development of the *Drosophila* CNS. Deregulation of Hippo signaling in neuroblasts causes a substantial increase in overall brain size. During normal CNS development, in postembryonic neurogenesis, Yki levels are high in the neuroepithelium of the larval CNS whereas it is low or absent in the medulla neuroblasts of the OL (Gailite et al., 2015; Poon et al., 2016). However, later in development, Yki overexpression in neuroblasts is sufficient to cause brain overgrowth (Reddy et al., 2010; Reddy and Irvine, 2011). A role for the Yki in control of NSC in the glioma brain, however, has not been reported.

Recently, CD109/ (Tep1 in *Drosophila*) was shown to be a novel upstream regulator of YAP/TAZ during glioma growth (Minata et al., 2019). CD109 belongs to the group of Thioester containing proteins (TEPs) which are involved in antimicrobial response in both vertebrate and invertebrate models, and raise inflammatory responses in vertebrates (Shokal et al., 2018). TEPs are classified into two subfamilies – complement factors and alpha-2 macroglobulins. Both complement factors and α-2 macroglobulins serve important functions in recognition as well as clearance of pathogens from the host. CD109 is a glycosylphosphatidylinositol-anchored glycoprotein and is a member of the α-2 macroglobulin/C3, C4, C5 family (Lin et al., 2002). High levels of CD109 have been reported in multiple cancers, including GBM (Hashimoto et al., 2004; Tao et al., 2014; Chuang et al., 2017; Shiraki et al., 2017). Mammalian studies have shown that CD109 is an oncogenic driver of tumor initiation and radioresistance in GBM. In addition, an important molecular link between, CD109, MES transition, and oncogenic signaling through Hippo pathway effectors yes-associated protein and transcriptional coactivator (YAP/TAZ) was identified (Minata et al., 2019). These studies showed that silencing CD109 reduced the total levels of YAP independent of the Hippo pathway. Further, CD109 was shown to act upstream of the YAP/TAZ signaling pathway and contribute to the stem cell and tumor-promoting properties of CD109-positive tumor cells.

Using the GAL4-UAS system in *Drosophila*, we have established a glioma model by coactivation of the PI3K and MAPK pathways [by downregulation of Pten (*UAS-Pten*^RNAi^) and overexpression of oncogenic Ras (*UAS-Ras*^V12^)] in the glial cells (*Repo-GAL4*) of the larval CNS (Waghmare et al., 2014; Cheng et al., 2016). The glial cells are tagged by the GFP reporter (*UAS-GFP*), and can be easily identified in the larval brain (Figure 1A). We previously showed that loss of the *Drosophila* homolog of CD109, Thioester containing protein 1 (Tep1) in glioma cells (*repo-Gal4> UAS-GFP*, *UAS-Pten*^RNAi^, *UAS-Ras*^V12^, *UAS-Tep1*^RNAi^) substantially reduced Yki levels and activity, and attenuated gliomagenesis *in-vivo* (Minata et al., 2019). However, if the Yki-Tep interaction regulates overall neuroblast numbers or organ size (size of the larval brain) remains unclear.

Here we describe a role for Yki in stem cell renewal during glioma growth in *Drosophila*. Our findings reveal that glioma grow due to the formation of a growth front at the edge of the neuropil region. Ectopic neuroblasts formed at this growth front contribute to the overall glioma growth. We further show that Tep1 downregulation specifically restricts this ectopic progression thereby restricting neuroblasts to the CB and reducing glioma growth. Moreover, we show that the glioma region of the CB shows no significant increase in Yki-mediated target gene expression (*ex-lacZ*, a Yki activity reporter) suggesting that in post-mitotic cells high levels of Yki activity is not maintained. However, Yki target genes like *Drosophila* Inhibitor of Apoptosis Protein 1 (DIAP1), and the Yki reporter *ex-lacZ* are upregulated at the edge of the growth front where ectopic neuroblasts and GMCs are being specified. Overall, we show a mechanism for glioma growth that depends on Yki and show that Tep1 acts upstream of Yki in this system.

## Materials and Methods

### Fly Strains

All fly mutants and transgenic lines are described in FlyBase, and were obtained from the Bloomington *Drosophila* Stock Center unless otherwise specified. The fly stocks used in this study are: *repoGal4 UAS-GFP/TM6B* (Waghmare et al., 2014), *UAS-Pten*^RNAi^ (BL 8548), *UAS-Ras*^V12^ (BL 5788), *UAS-Tep1*^RNAi^ (BL 32856), *ex-lacZ* (a gift from Georg Halder), *UAS-Yki*^RNAi^ (BL 34067). All the *Drosophila* fly stocks and crosses were maintained at 25°C on standard cornmeal, molasses, and agar medium. To induce glioma *UAS-Pten*^RNAi^, *UAS-Ras*^V12^ female virgins were outcrossed to *repoGal4 UAS-GFP/TM6B* males. The non-TM6B F1 larvae were the genotypic class representing the experimental group and were selected for further study. The genetic crossing scheme resulted in the induction of brain neoplasms (glioma) in both F1 male and female larvae. A comparison of frequency and size of the glioma brains showed no significant difference based on sex of the larva. To study effects of Tep downregulation, *UAS-Pten*^RNAi^; *UAS-Ras*^V12^; *UAS-Tep1*^RNAi^/*TM6B* virgin females were crossed to *repoGal4 UAS-GFP/TM6B* males, and non-TM6B larvae selected for experiments. To study *ex-lacZ* reporter expression in larval CNS, *ex-lacZ/CyO*; *repoGAL4 UAS-GFP/TM6B* flies were generated and outcrossed virgins of appropriate genotypes.

### Immunohistochemistry

Standard protocol was followed to prepare samples for immunohistochemistry (Kango-Singh et al., 2002). Briefly, third instar larval brains were dissected in PBS and fixed for 20 min in 4% paraformaldehyde. The samples were washed in PBST (PBS+0.4% Triton-X-100), blocked in PBSTN (PBST + 2% Normal donkey serum) and incubated at 4^o^C overnight in primary antibodies at appropriate dilution. The samples were then washed three times in PBST and processed for immunohistochemistry by incubation in appropriate secondary antibodies for 2 h, washed twice in PBST, mounted in VectaShield (Vector Labs), and scanned using Laser Scanning Confocal Microscopy (Olympus Fluoview 1000, 3000) at 20× magnification. The primary antibodies used were mouse anti-Prospero (DHSB, 1:100), rat anti-Miranda (Ab-Cam, 1:200), mouse anti-PH3 (1:250, DSHB), mouse anti-DIAP1 (1:100, gift of B. Hay), mouse anti-β-galactosidase (1:100, DHSB), rabbit anti-β-galactosidase (1:100, DHSB). Secondary antibody used were donkey Cy3-conjugated anti-mouse IgG (1:200, Jackson ImmunoResearch), donkey Cy3-conjugated anti-rabbit IgG (1:200, Jackson ImmunoResearch) and donkey Cy5-conjugated anti-rat IgG (1:250, Jackson ImmunoResearch).

### Image Analysis

**FIGURE 2:**
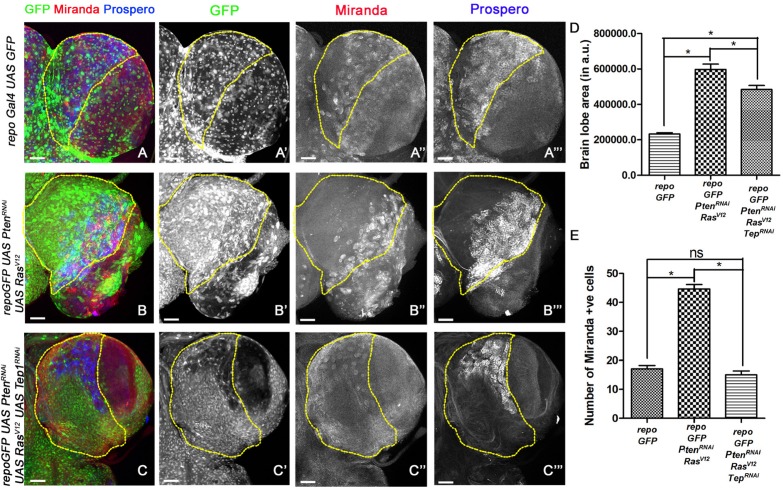
Loss of Tep1 suppresses ectopic Neuroblasts that drive glioma growth. Panels show comparisons of Miranda (red) and Prospero (blue) positive cells in the central brain of *Drosophila* larval CNS from third instar larvae are shown for the following genotypes **(A–C”’)** GFP (green, glia), Mira (red, gray) and Pros (blue, gray) expression in third-instar larval CNS from *repo-GFP* **(A–A”’)**, *repo*-GFP>*UAS-Pten*^RNAi^; *UAS-Ras*^V12^ **(B–B”’)**, *repo*-GFP>*UAS-Pten*^RNAi^; *UAS-Ras*^V12^; *UAS-Tep1*^RNAi^ **(C–C”’)**. **(D)** Quantification of brain lobe size was done using the magnetic lasso tool in Photoshop to create an ROI for measuring pixel values. At least five independent biological replicates for each genotype were compared. **(E)** Quantification of number of Mira positive cells in the central brain is shown in the graph. For **(D)** and **(E)** unpaired 2-tailed *T*-test with *n* = 5, 95% confidence was performed using GraphPad Prism 5, *p* < 0.05. Yellow ROI boxes indicate area in which Mira +ve cells are counted. All images were scanned at identical magnification [20×, scale bars, 100 μm = 62px].

Brain lobes from both males and female larvae were analyzed as no sex-specific differences were observed. The images collected from the confocal microscope were quantified using the measurement log function of Photoshop (Adobe Photoshop CC 2018). The ROI used for quantification is shown by yellow lines in each figure. The number of PH3 (Figure 1) and Miranda (Figures 2, 5) positive cells were calculated using the count function in the central brain region marked by boxed ROI’s. Please note no cells from the OL were included in these counts. Mean gray value was calculated for Diap1 (Figure 4) expression by creating 3 square ROIs of 50^∗^50 square pixels in the CB region. A 2-tailed unpaired *T*-test and Mann–Whitney *U* test was performed to check whether the observed differences were significant (*p* < 0.05) using GraphPad Prism 5 software or MS Excel 2016. Error bars in graphs are defined in the figure legends and represent the mean ± SD (standard deviation). Sample number is denoted by ‘n’ in figure legends, and *n* = 5 for all genotype.

**FIGURE 3:**
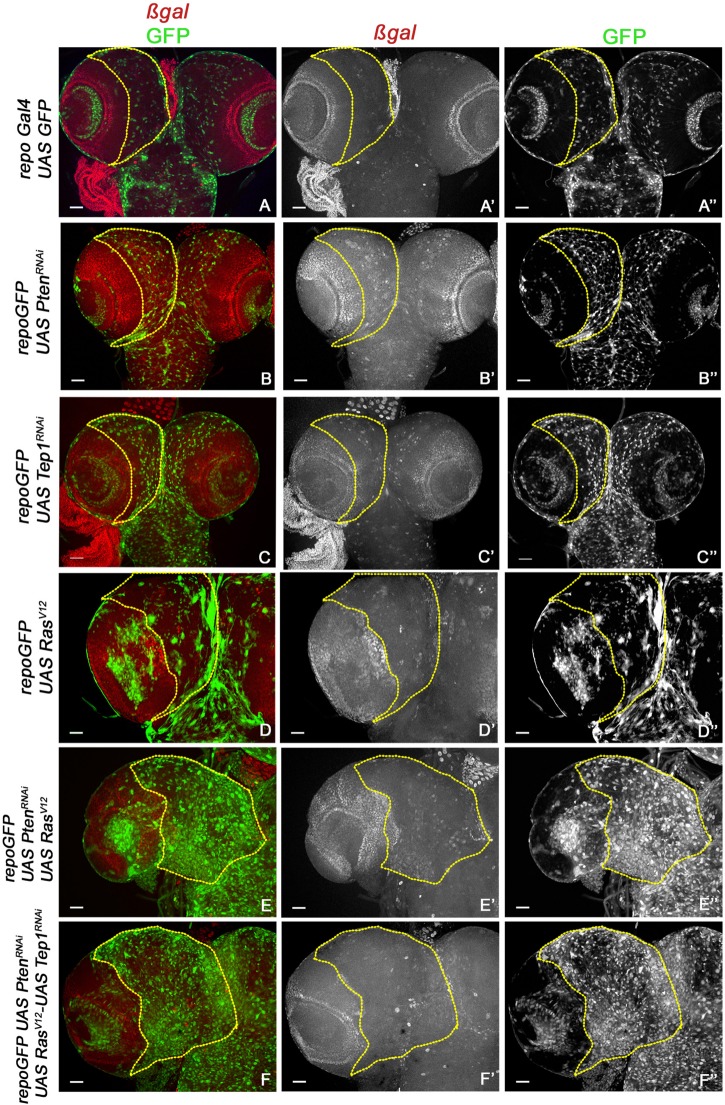
Yki reporter activity in glioma brains. A comparison of Yki reporter activity is shown. **(A–F”)** show expression of *ex-lacZ (ß-*gal, red) in larval brains. Glia are marked by GFP (green) in the following genotypes: *repo-GFP* **(A–A”)**, *repo*-GFP>*UAS-Pten*^RNAi^ **(B–B”)**, *repo*-GFP>*UAS-Tep*^RNAi^ **(C–C”)**, *repo*-GFP>*UAS-Ras*^V12^ **(D–D”)**, *repo*-GFP>*UAS-Pten*^RNAi^; *UAS-Ras*^V12^ **(E–E”)**, *repo*-GFP>*UAS-Pten*^RNAi^; *UAS-Ras*^V12^; *UAS-Tep1*^RNAi^ **(F–F”)**. Brain lobes are imaged at 20× magnification with scale bars 100 μm = 62px. Yellow lines represent the ROI within which Yki reporter activity is compared.

**FIGURE 4:**
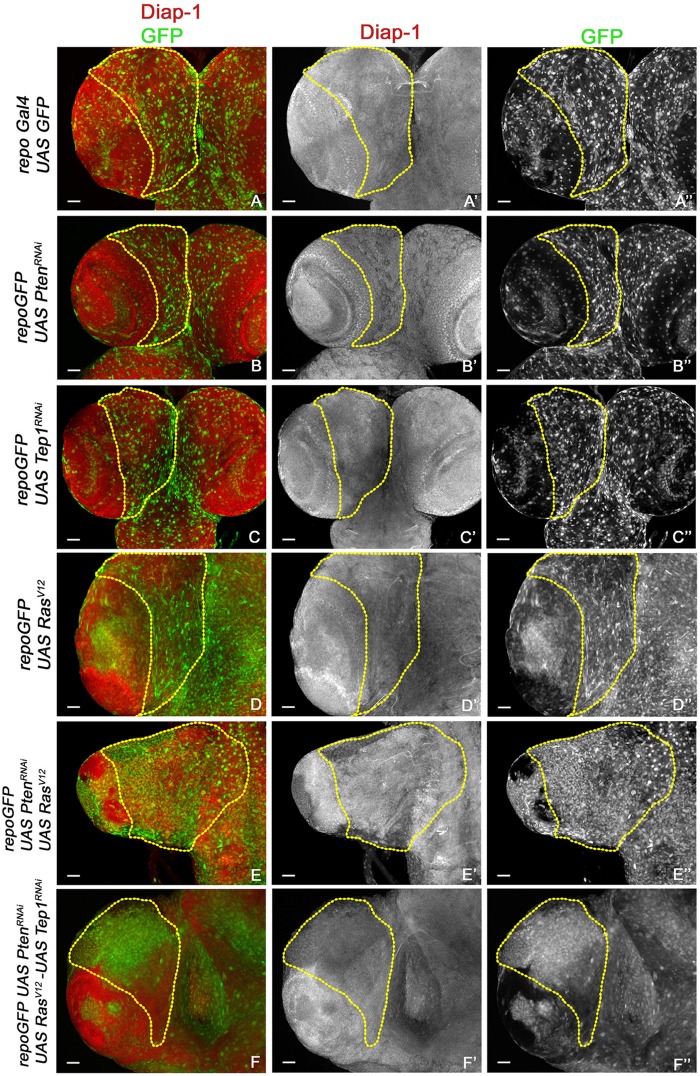
Increased survival in glioma. **(A–F”)** Expression of Diap1 (red) and glial cells marked with GFP (green) in third-instar larval CNS from *repo*-GFP **(A–A”)**, *repo*-GFP>UAS-*Pten*^RNAi^ **(B–B”)**, *repo*-GFP>UAS-*Tep*^RNAi^ **(C–C”)**, *repo*-GFP>*UAS-Ras*^V12^ **(D–D”)**, *repo*-GFP>*UAS-Pten*^RNAi^; *UAS-Ras*^V12^ **(E–E”)**, *repo*-GFP>*UAS-Pten*^RNAi^; *UAS-Tep*^RNAi^/*UAS-Ras*^V12^ **(F–F”)**. Brain lobes are imaged at 20× magnification with scale bars 100 μm = 62px.

**FIGURE 5:**
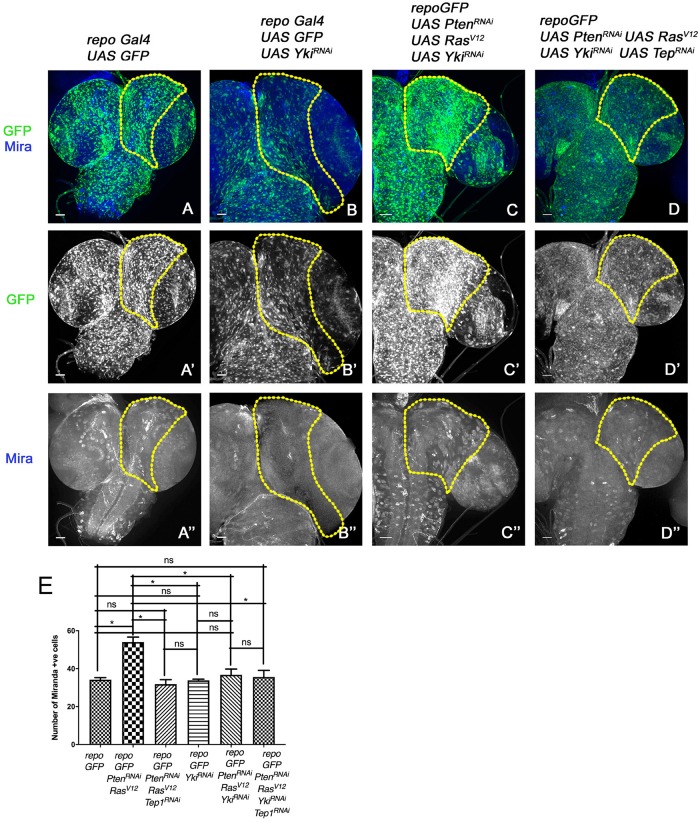
Downregulation of Yki reduces glioma growth and neuroblast number in larval CNS. **(A–D”)** panels show a comparison of neuroblast number in the central brain region (the yellow line marks the ROI in one brain lobe for each genotype). In all panels glia are marked by GFP (green, gray), and neuroblasts by Mira expression (blue, gray). The genotypes are: *repo*-GFP **(A–A”)**, *repo*-GFP>*UAS-Yki*^RNAi^ **(B–B”)**, *repo*-GFP>*UAS-Pten*^RNAi^; *UAS-Ras*^V12^; *UAS-Yki*^RNAi^ **(C–C”)**, and *repo*-GFP>*UAS-Pten*^RNAi^; *UAS-Ras*^V12^; *UAS-Yki*^RNAi^/*UAS-Tep*^RNAi^ **(D–D”)**. **(E)** Quantification of number of Mira positive cells in the central brain region is shown for the indicated genotypes. Unpaired 2-tailed *T*-test with *n* = 5, 95% confidence was performed using GraphPad Prism 8, *p* < 0.05. Yellow ROI boxes indicate area in which Mira positive cells are counted. All images were scanned at identical magnification [20×, scale bars, 100 μm = 62px].

## Results

### Downregulation of Tep1 Reduces Proliferation in Glioma

The size of the developing CNS depends on the proliferative potential of NSCs, due to the finite progeny produced by this cell type (Sousa-Nunes et al., 2010; Egger et al., 2011; Homem and Knoblich, 2012). Therefore, we first tested cell proliferation in the glioma model by assessing expression of Phospho-histone H3 (PH3, red, gray in Figure 1) which marks mitotic figures (Su et al., 1998). For our studies, we quantified levels of PH3 in the central brain region (ROI marked by yellow dashed line in each panel). In the wild type (WT) brain (Figure 1A), the mitotic figures are predominantly seen in the optic lobe (Figure 1A”), in contrast, fewer mitotic figures are found in the central brain region where a large number of differentiated glial cells are present (Figures 1A,D). We compared these to larval brains from *Drosophila* glioma bearing larvae. The glioma were induced by co-activation of oncogenic Ras and the PI3K pathway by the expression of *UAS-Pten*^RNAi^; *UAS-Ras*^V12^ under the glia specific Repo-Gal4 driver (henceforth referred to as *repoGal4>UAS-Pten*^RNAi^; *UAS-Ras*^V12^; *UAS-GFP*). The resulting glioma showed an increased number of mitotic figures in the central brain (Figures 1B,D). Next, we tested the effect of downregulation of Tep1 on proliferation in the central brain in *repoGal4>UAS-Pten*^RNAi^; *UAS-Ras*^V12^; *UAS-GFP/UAS Tep1*^RNAi^ (Figure 1C). Interestingly, we noticed a significant downregulation in mitotic figures in the central brain region (Figures 1C,D). Of note, all three genotypes showed a high density of mitotic figures in the region in the optic lobe (Figures 1A”–C”, marked by yellow arrows). Taken together, these data suggest that the coactivation of PI3K and oncogenic Ras cause increased cell proliferation leading to gliomagenesis in the central brain. Further, reduction in the glioma size by downregulation of Tep1 likely occurs through downregulation of cell proliferation. So next, we tested if Tep1 was involved in growth or differentiation of NSCs in our glioma model.

### Glioma Grow by Specification of Ectopic Neuroblasts

The larval CNS is comprised of mostly post-mitotic cells like glia and neurons (Lee et al., 1999; Pearson and Doe, 2003). To understand the nature of cells that contribute to glioma growth, we tested the expression of Miranda (Mira), a key component of neuroblast differentiation in the larval CNS (Wang et al., 2006; Sousa-Nunes et al., 2010). Neural stem cells/neuroblasts divide by a tightly regulated process to produce the correct number and type of neurons (Homem and Knoblich, 2012; Sousa-Nunes and Somers, 2013). Mira (Figure 2, red, gray) is expressed in the asymmetrically dividing neuroblasts which gives rise to two daughter cells – a neuroblast and GMC. The GMCs express specific transcription factors in order to divide and form two differentiated cells, neurons and glia. One of the key markers for GMCs in the larval CNS is Prospero (Pros, blue, gray) (Figure 2). To assess if deregulation NSC proliferation is associated with glioma phenotypes, we compared NSC numbers and overall brain lobe size in age matched brains wild-type larvae and larvae where glioma was induced (Figure 2). We marked NSCs by Mira (Figure 2, blue, gray) and GMCs by Pros (Figure 2, red, gray) and compared larval brain from wild type to glioma brains. Compared to wild-type (*repoGal4>UAS-GFP*), significantly more Miranda positive cells were observed in the glioma model [*repoGal4>UAS-Pten*^RNAi^; *UAS-Ras*^V12^; *UAS-GFP*] (marked by yellow ROIs) (Figures 2A,A”’,B,B”’, quantified in E). Consistent with this observation, Pros expression was also deregulated in the glioma brain (Figures 2A”,B”). Interestingly, in the glioma brain, the domain of Mira and Pros expressing cells seems to undergo a lateral shift at the edge of the central brain abutting the optic lobe (Figures 2B–B”’). Compared to WT, a significant overgrowth is seen in brain lobes in the *repoGal4>UAS-Pten*^RNAi^; *UAS-Ras*^V12^; *UAS-GFP* glioma (Figure 2D). Concomitantly, the number of Mira positive cells is also significantly higher in the glioma brain as compared to WT (Figure 2E). In summary, in glioma, the number of stem cells and their domain of expression is altered compared to WT. This shift in the expression pattern may account for the elongated appearance of the brain lobes as the presence of ectopic neuroblasts promotes differentiation of supernumerary GMC, neurons and glia, and overall promote glioma growth.

### Downregulation of Tep Reduced Neuroblast Number and Glioma Size

When Tep1 is downregulated in glioma (*repoGal4>UAS-Pten*^RNAi^; *UAS-Ras*^V12^; *UAS-GFP/UAS-Tep1*^RNAi^), both Mira (Figures 2C,C”) and Pros (Figures 2C,C”’) expression domains are restricted back to the central brain region. Furthermore, there is significant reduction in the size of the glioma brains (Figure 2D). However, the brain size was not completely restored to WT (Figure 2D). Moreover, in *repoGal4>UAS-Pten*^RNAi^; *UAS-Ras*^V12^; *UAS-GFP/UAS-Tep1*^RNAi^ glioma, the number of Mira positive NSCs is restored to near WT in the central brain region (Figure 2E). Collectively, these data suggest that Tep1 is required in the growth and differentiation of NSCs, and loss of Tep1 in glioma leads to reduction of glioma growth by reducing neuroblast number.

### Yki Activity Affects Glioma Growth

Previous studies have established a role for Hippo signaling in controlling proliferation potential of neuroblasts, and for normal glial growth during nervous system development (Reddy and Irvine, 2011; Poon et al., 2016). To test the role of Yki misregulation in glioma, we first monitored changes in expression of the reporter *ex-lacZ* (Figure 3, red, gray) as a direct measure for Yki activity. In the WT control *repoGal4>UAS-GFP*, *ex-lacZ* is ubiquitously expressed with a prominent stripe of expression in the optic lobe (Figures 3A,A”). In *repoGal4>UAS-Pten*^RNAi^; *UAS-GFP* (Figures 1B,B’) and *repoGal4>UAS-Tep1*^RNAi^; *UAS-GFP* (Figures 1C,C’) no significant change is seen in either *ex-lacZ* expression or the number of glia (GFP positive, Figures 3B”,C”). This suggests that activation of PI3K signaling or loss of Tep1, in Repo driven cells, does not affect the overall size of *Drosophila* larval CNS. Similarly, *ex-lacZ* expression is not significantly increased when oncogenic *Ras*^V12^ (*repoGal4>UAS-Ras*^V12^; *UAS-GFP*, Figures 1D,D’) is expressed (Figure 3D”). Interestingly, in *repoGal4>UAS-Pten*^RNAi^;*UAS-Ras*^V12^ glioma *ex-lacZ* expression is enhanced in the optic lobe but not within the central brain (Figures 3E,E’). This is consistent with previous reports that Yki activity is high in proliferating neuroepithelium but not in the post-mitotic glial cells. However, when Tep1 is downregulated in the glioma (*repoGal4>UAS-Pten*^RNAi^; *UAS-Ras*^V12^; *UAS-GFP/UAS-Tep1*^RNAi^), *ex-lacZ* expression as well as the number of glia are reduced (Figures 3F–F”). We also looked at Diap1 levels as a second measure of Yki activity in the central brain region. Diap1 is a transcriptional target of the Hippo pathway, and increased Diap1 levels are correlated with increased cell survival (Figure 4). As compared to WT (*repoGal4>UAS-GFP*, Figures 4A,A’), there is no significant change in Diap1 level upon loss of Pten (*repoGal4>UAS-Pten*^RNAi^; *UAS-GFP* Figures 4B,B’), loss of Tep (*repoGal4>UAS-Tep1*^RNAi^; *UAS-GFP*, Figures 4C,C’), and overexpression of Ras (*repoGal4> UAS-Ras*^V12^; *UAS-GFP*, Figures 4D,D’). In contrast, DIAP1 levels were increased in the glioma brain (*repoGal4>UAS-Pten*^RNAi^; *UAS-Ras*^V12^; *UAS-GFP*, Figures 4E,E’) and downregulated in *repoGal4>UAS-Pten*^RNAi^; *UAS-Ras*^V12^; *UAS-GFP/UAS-Tep1*^RNAi^ (Figures 4F,F’). Thus, Yki activity is high in the glial precursors and in the neuroepithelium, but not in terminally differentiated cells like glia. Overall, these data suggest that modulating Yki levels by depletion or overexpression may provide better insights about the role of Yki in glia and NSC proliferation.

### Downregulation of Yki Results in Decreased Glioma Growth Due Decreased Neuroblast Proliferation

To understand if the Tep1-dependent effects occur through Yki, we downregulated Yki (*repoGal4>UAS-Pten*^RNAi^; *UAS-Ras*^V12^; *UAS-GFP/UAS-Yki*^RNAi^) in the glioma model (Figure 5). Compared to WT (*repoGal4> UAS-GFP*, Figure 5A), downregulation of Yki (*repoGal4> UAS-GFP/UAS-Yki*^RNAi^ resulted in a significant reduction in number of Mira positive NSCs (Figure 5B, quantified in E). Yki downregulation in glioma (*repoGal4>UAS-Pten*^RNAi^; *UAS-Ras*^V12^; *UAS-GFP/UAS-Yki*^RNAi^, Figure 5C) showed a distinct reduction in brain size (Figure 5C). Interestingly, in these brains, the neuroblasts were restricted back to the central brain region (Figure 5C”). These phenotypes are reminiscent of the effects of downregulation of Tep1 (*repoGal4>UAS-Pten*^RNAi^; *UAS-Ras*^V12^; *UAS-GFP/UAS-Tep1*^RNAi^, Figures 2C–C”’) both in terms of reduction in Yki activity (Figure 4F) and the number of NSCs. These data are consistent with our recent findings that Tep1 acts upstream of Yki and influences glioma growth (Minata et al., 2019).

In order to test the interactions between Tep and Yki in the context of glioma growth, we wanted to examine if overexpression of Yki could rescue the effects of loss of Tep1 in our glioma model using *repoGAL4*. However, Yki overexpression in glia (*repoGal4>UAS-GFP/UAS-Yki*) caused lethality in early second instar larval stage, limiting our ability to perform a genetic epistasis experiment. Given the lack of tools for Tep1 overexpression, and our previous data suggesting a downstream role for Yki, we compared the effects of loss of Tep1 or Yki or both in the glioma model. We compared the overall brain size; and the number and location of Mira-positive neuroblasts as a measure for this comparison (Figure 5). The simultaneous loss of Tep and Yki (*repoGal4>UAS-Pten*^RNAi^; *UAS-Ras*^V12^; *UAS-GFP/UAS-Yki*^RNAi^; *UAS-Tep1*^RNAi^, Figures 5D–D”) showed reduction in brain size, and the number and location of Mira-positive cells. Although these results show an effect that resembles loss of *yki*, it does not exclude the possibility that Yki and Tep may act via a convergent synergistic interaction.

## Discussion

Glioma are a diverse and heterogeneous group of cancers that affect the brain and the spinal cord, and account for 80% of all malignant primary CNS tumors (Ho et al., 2014; Ostrom et al., 2018). Genomic studies have characterized the molecular and genetic drivers and epigenetic networks associated with glial neoplasms. Amongst the different brain tumors, Glioblastoma multiforme (GBM) is the most aggressive and lethal glial neoplasm. The growth of GBM is attributed to glioma stem cells (GSCs), which is a small population of GBM cells that contribute to gliomagenesis, chemotherapy resistance, and tumor relapse (Modrek et al., 2014; Yin et al., 2014; Liebelt et al., 2016). The GSCs share many phenotypic and functional similarities to the NSCs, e.g., in response to molecular signals GSCs undergo self-renewal and generate cells with neuron- or glia-like properties. Once GSCs differentiate they lose their self-renewal property but can acquire stemness (Flavahan et al., 2013). There is emerging evidence that GBM arises from NSCs or glial precursor cell in patients as well (Kawamura et al., 2018; Lee et al., 2018).

The development of Drosophila CNS and its constituent cell-types is well-established (Knoblich, 2001; Sousa-Nunes et al., 2010; Doe, 2017). The presence of analogous cell types like the NSCs, glia or neurons in *Drosophila* allows for comparison of findings between different preclinical models (Read, 2011). The Hippo pathway effector Yki is a key regulator of neural development (Kawamori et al., 2011; Read, 2011; Poon et al., 2016; Lavado et al., 2018). During CNS development, the NSCs originate from the neuroepithelium. In the central brain and ventral nerve cord NSCs delaminate during embryonic development whereas in the optic lobe NSCs remain neuroepithelial until the larval stages, when Hippo and Notch pathways regulate the epithelial to NSC transition (Reddy et al., 2010; Gailite et al., 2015). The Hippo pathway is also known to affect proliferative potential of neuroblasts and brain size by affecting cell-cycle speed and size of NSCs, and the overall developmental timing of NSC proliferation (Poon et al., 2016).

We identified a role of NSCs in the growth of glioma induced by coactivation of PI3K and Ras/MAPK signaling in Drosophila. The glioma grows due to the formation of an interface at the edge of the neuropil region where supernumerary neuroblasts (Figure 2) are specified that lead to differentiation of supernumerary GMC, glia, and neurons. The stochastic coactivation of PI3K and MAPK/EGFR pathways promotes the formation and progression of this growth front causing the brain lobes to appear elongated and misshapen ultimately causing lethal neoplasms.

We further show that the recently identified Yki modifier, Tep1 (Drosophila ortholog of CD109) is a modifier of glioma phenotype due to its effect on NSC number (Figure 2). Our study also demonstrates that the Tep-Yki interaction plays an important role in glioma growth due to the effect of Tep1 on Yki-mediated neuroblast proliferation. The reduction in the neuroblast number may be attributed to the effects of Tep1 downregulation, which compromises Yki mediated stem cell function in glioma thereby identifying a new upstream regulator of Yki in the larval CNS (Figures 3–5). The exact mechanisms by which Tep1 interacts with Yki remains to be defined. However, we have preliminary evidence from experiments in imaginal disc development suggesting that Tep1-Yki interaction is not limited to the CNS but may occur in epithelial cells as well. Furthermore, other components of Hippo pathway, e.g., the Hippo (Hpo) kinase- an upstream regulator of Yki may be part of this interaction. The similarity of glioma reduction phenotypes of downregulation of Tep1 and downregulation of Yki or both suggests that Tep1 may act through Yki and the Hippo pathway to regulate NSC number, or that Tep1and Yki synergistically regulate NSC number in Drosophila larval brain. In the future, it would be interesting to identify the mechanisms by which Tep1 interacts with Yki to regulate neuroblast number in larval CNS during gliomagenesis. Given the conservation of gene functions, *Drosophila* glioma models may prove informative in further understanding the Tep (CD109) and Yki (YAP/TAZ) in tumor growth and progression. These studies are especially interesting as the Tep1 mammalian ortholog, CD109 is known to be an oncogenic driver of tumor initiation in mammalian cancer.

## Data Availability Statement

The raw data supporting the conclusions of this article will be made available by the authors, without undue reservation, to any qualified researcher.

## Author Contributions

KG, KS, and MK-S designed the experiments, wrote and edited the manuscript. KG and KS performed the research and analyzed the data. All authors contributed to manuscript revision, read and approved the submitted version.

## Conflict of Interest

The authors declare that the research was conducted in the absence of any commercial or financial relationships that could be construed as a potential conflict of interest.
